# A Randomised Controlled Trial Comparing Thermoformed and 3D-Printed Retainers in Young Adults: Evaluation of Post-treatment Stability and Patient Satisfaction

**DOI:** 10.1007/s00784-026-06793-z

**Published:** 2026-03-17

**Authors:** Hui Shan Boo, Norhidayah Nor zahidah Mohd Tahir, Aufa Dahlia Bahar, Simon J. Littlewood, Saritha Sivarajan

**Affiliations:** 1 Department of Paediatric Dentistry and Orthodontics, Faculty of Dentistry, Universiti Malaya, Lembah Pantai, Kuala Lumpur, 50603 Malaysia; 2 Department of Orthodontics, St Luke’s Hospital, Bradford, UK

**Keywords:** 3D-printed retainers, Thermoformed retainers, Retention, Post-treatment stability, OHRQoL

## Abstract

**Introduction:**

To compare post-treatment stability and oral health–related quality of life (OHRQoL) in young adults wearing thermoformed retainers (TFR) versus direct 3D-printed retainers (3DPR) over a six-month part-time retention period.

**Materials and methods:**

This single-centre, two-arm parallel randomised controlled trial allocated 30 debonded orthodontic patients (≥ 18 years) to either TFR (Erkodur PETG, 1.0 mm) or 3DPR (NextDent Ortho Flex, 0.75 mm) in a 1:1 ratio. All retainers were worn part-time (8–12 h/day). Digital intraoral scans were obtained at baseline (T0) and six months (T2) using Trios 3, and post-treatment stability was assessed digitally using Little’s Irregularity Index, intercanine width, intermolar width, arch length, overjet, and overbite. OHRQoL was evaluated at both time points using the OHIP-14(M) questionnaire. Non-parametric tests were applied following the Shapiro–Wilk assessment, and analyses were conducted using an intention-to-treat approach.

**Results:**

One dropout in the 3DPR group at T2. At T2, no significant between-group differences were found in Little’s Irregularity Index, intercanine width, intermolar width, arch length, or overjet (*p* > 0.05). Overbite values were statistically higher in the TFR group at T2 (*p* < 0.05); however, the difference was not clinically significant. Changes in post-treatment stability from T0 to T2 (ΔT2–T0) did not differ significantly between groups (*p* > 0.05). Total OHIP-14(M) scores improved significantly from T0 to T2 in both groups (TFR *p* = 0.016; 3DPR *p* = 0.013), with no significant between-group differences at either time point (*p* > 0.05). A small but significant difference was observed in the psychological disability domain at T2, with slightly higher scores in the 3DPR group (*p* = 0.020). No harm was observed.

**Conclusions:**

TFR and 3DPR demonstrated comparable post-treatment stability and similar improvements in OHRQoL after six months of part-time wear.

**Registration:**

ClinicalTrials.gov (Identifier NCT05968625).

Protocol: The protocol was published before trial commencement

**Supplementary Information:**

The online version contains supplementary material available at 10.1007/s00784-026-06793-z.

## Introduction

Prolonged retention with part-time or full-time retainer wear is crucial in preventing relapse following orthodontic treatment [1]. Long-term studies have demonstrated that mandibular incisor alignment continues to change for decades after treatment, irrespective of the orthodontic appliance used or treatment modality [2, 3]. These findings emphasise that post-treatment stability is not a static outcome but a lifelong concern. The biological basis of relapse, including periodontal and gingival fibre reorganisation and neuromuscular adaptation, further supports the necessity for long-term or permanent retention in many patients [4, 5]. Most patients require long-term retention to maintain their corrected tooth positions, and post-treatment stability is now recognised as a key indicator of overall orthodontic treatment quality [6]. The retention phase allows reorganisation of gingival and periodontal tissues and adaptation of neuromuscular structures, while limiting changes associated with ongoing dentofacial growth [7, 8].

Thermoformed retainers (TFR) are widely prescribed due to their favourable aesthetics, cost-effectiveness, and high patient acceptance compared with traditional Hawley retainers during the initial retention phase [9, 10]. Evidence from randomised controlled trials has demonstrated that TFRs provide comparable, and in some cases superior, maintenance of incisor alignment when compared with Hawley retainers, particularly in the mandibular arch [11]. Importantly, Hichens et al. reported significantly higher patient satisfaction, comfort, and acceptance with TFRs, which were associated with improved compliance despite their inferior durability compared with Hawley retainers [9]. A recent Cochrane systematic review by Martin et al. (2023) found that, when comparing TFR and Hawley retainers, part-time Hawley retainers exhibited lower tooth stability and poorer patient satisfaction than TFR, despite having superior durability [12]. There is no difference in stability between different wearing regimes (part-time or full-time) for either type of retainer [12].

Rapid advancements in digital dentistry have transformed orthodontic practice. The utilisation of intra-oral scanning and three-dimensional (3D) printing aims to enhance clinical efficiency, precision, and sustainability [13, 14]. The direct 3D printing of orthodontic appliances, including retainers, removes intermediary steps such as model fabrication and thermoforming, thereby streamlining laboratory procedures and potentially decreasing material waste [14]. In contrast to thermoforming, digital workflows allow appliance characteristics to be prescribed at the design stage, shifting control from a process-dependent to a design-driven manufacturing approach.

Unlike thermoformed retainers, which are characterised by unpredictable material thinning during heating and forming, direct 3D-printed retainers are engineered with a digitally defined, uniform thickness [15, 16]. Edelmann et al. demonstrated that 3D-printed orthodontic appliances exhibit increased thickness with potential implications for appliance rigidity, perceived bulk, and fit during prolonged intra-oral wear [16]. Jindal et al. further showed that direct 3D-printed orthodontic appliances demonstrate superior geometric accuracy and greater mechanical stiffness compared with conventional thermoformed appliances, largely due to the additive manufacturing process and post-curing conditions [17].

Although these mechanical characteristics may improve accuracy and structural stability, the associated increases in thickness and rigidity may adversely affect patient comfort and intraoral tolerance during daily wear [18–20]. Importantly, Jindal et al. cautioned that in vitro mechanical and geometric advantages do not necessarily translate into clinical acceptability, highlighting the need to evaluate patient-reported outcomes [17]. Therefore, assessment of patient perception and acceptability is crucial to establish whether the material and mechanical advantages of 3D-printed retainers are experienced positively by patients during long-term wear.

Since the success of retainers depends significantly on patient compliance, it is crucial to understand patients’ perceptions regarding the impact on oral health [21]. Evaluating patient-reported outcomes serves as an important complement to traditional measures of post-treatment stability [1, 22]. Instruments assessing oral health-related quality of life (OHRQoL) address functional limitations, discomfort, psychological effects, and social adaptation elements not captured by occlusal measurements alone [23, 24]. Variations in retainer thickness, flexibility, and appliance awareness may influence the patient experience and their willingness to wear the device, thereby indirectly affecting retention success.

Although OHRQoL has been extensively studied during active orthodontic treatment and after appliance removal [25, 26], there remains a paucity of evidence on patient-reported outcomes during the retention phase, especially when comparing retainers produced through digital versus conventional workflows.

Therefore, this randomised controlled trial aimed to compare post-treatment stability and oral health-related quality of life in young adults wearing either thermoformed retainers or direct 3D-printed retainers over a 6-month retention period. The primary outcome is post-treatment stability between TFR and 3DPR baseline (T0) and 6 months (T2). The secondary outcome is the OHRQoL of patients wearing TFR and 3DPR at T0 and T2. The null hypothesis is that there are no significant differences in post-treatment stability and OHRQoL between TFR and 3DPR at all time points and time intervals (T0-T2).

## Materials and methods

### Trial design and ethical approval

This study was a single-centre, two-arm, parallel, single-blinded, prospective, randomised controlled trial with a 1:1 allocation ratio. The research was conducted at the Postgraduate Orthodontic Clinic, Dental Specialists and Research Tower, Faculty of Dentistry at Universiti Malaya, from April 2023 to September 2024. The study protocol was approved by the Medical Ethics Committee at the Faculty of Dentistry, Universiti Malaya, prior to the initiation of the study (Reference: DF CD 2316/0036 (L)), with approval granted on 15th February 2023. In accordance with ethical standards, this clinical trial was registered with ClinicalTrials.gov (Identifier: NCT05968625).

### Participants, eligibility criteria, and settings

Patients scheduled for the debonding of orthodontic fixed appliances at the Faculty of Dentistry, Universiti Malaya, who met the eligibility criteria, were invited to participate in the study and were consecutively recruited between April 2023 and September 2023. The inclusion and exclusion criteria are listed below.

Inclusion criteria:

1. Adults aged 18 and over.
2. Completion of comprehensive fixed appliance treatment in both arches, followed by planned retention with TFR as specified in the original treatment plan. Retainer prescription was determined at the treatment planning stage based on routine clinical considerations and was not modified for the purposes of this study.
3. No intention to relocate within the study period; able to attend the three-monthly review appointments for six months.

Exclusion criteria:

1. Single-arch or sectional fixed appliances.
2. Spaced dentition or hypodontia requires tooth replacement on the retainer as a temporary measure.
3. Previous treatment with maxillary expansion.
4. Indicated for a fixed retainer or double retention regime (such as TFRs fitted over fixed retainers).
5. Premature debond from the original fixed appliances course.
6. Patient with a known history of bruxism.
7. Cleft lip and/or palate, or orthognathic cases.
8. Learning difficulties and inability to read written instructions or questionnaires in English or Malay.

Patient information sheets and consent forms were issued to the subjects who fulfilled the criteria. All patients provided written consent to participate before the debonding procedure. All collected data and information were handled with strict confidentiality and were only accessible to the research team members.

### Sample size calculation

The sample size calculation was based on a study by Kumar & Bansal (2011), which compared the effectiveness of two different removable retainers on post-treatment stability [27]. The sample size calculation was performed using G*Power version 3.1.9.7. Based on an effect size of 1.127, α = 0.05, and power = 0.80, a total of 22 subjects were needed to compare the two groups (11 per group). The final sample size was determined after accounting for a 30% dropout rate due to poor compliance with retainer wear [24], yielding 15 subjects per group. Assuming a within‑group standard deviation of approximately 1.5–1.8 mm for transverse dental width measures, this sample size provides 80% power to detect a between‑group difference of around 1.5–2.0 mm, which is considered clinically meaningful for post‑treatment stability outcomes. Since the 3D‑printed material tested was novel, a modest group size was also considered ethically appropriate to enable close monitoring of any adverse effects while still yielding robust preliminary estimates of clinical performance.

### Randomization and allocation concealment

Block randomization was conducted by an independent researcher (SS) who was not involved in the treatment, data collection, or data analysis phases of the study. This randomization employed a 1:1 allocation ratio with a block size of 6, resulting in a total sample size of 30. The randomization sequence was generated using an online tool (https://www.sealedenvelope.com*).*

The random allocation sequence was securely maintained in sealed, opaque envelopes, which were numbered sequentially. The principal investigator (BHS) remained unaware of the randomization allocation sequence throughout the process. Once subjects met the inclusion criteria and provided their consent to participate, the envelopes were opened, and the allocation was revealed to the operator on the day of the debond appointment.

### Interventions

All subjects underwent a standardised debond process, including the removal of bands and brackets by one clinician (BHS) using appropriate pliers, followed by composite removal with a tungsten carbide bur, scaling, and polishing. Standard post-treatment records include intraoral and extraoral photos, and alginate impressions (Hydrogum 5; Zhermack, Italy) were taken. Following appliance removal, all participants were issued an initial non-interventional TFR as part of routine clinical care to prevent immediate relapse while the allocated study retainers were fabricated.

**Fig. 1:**
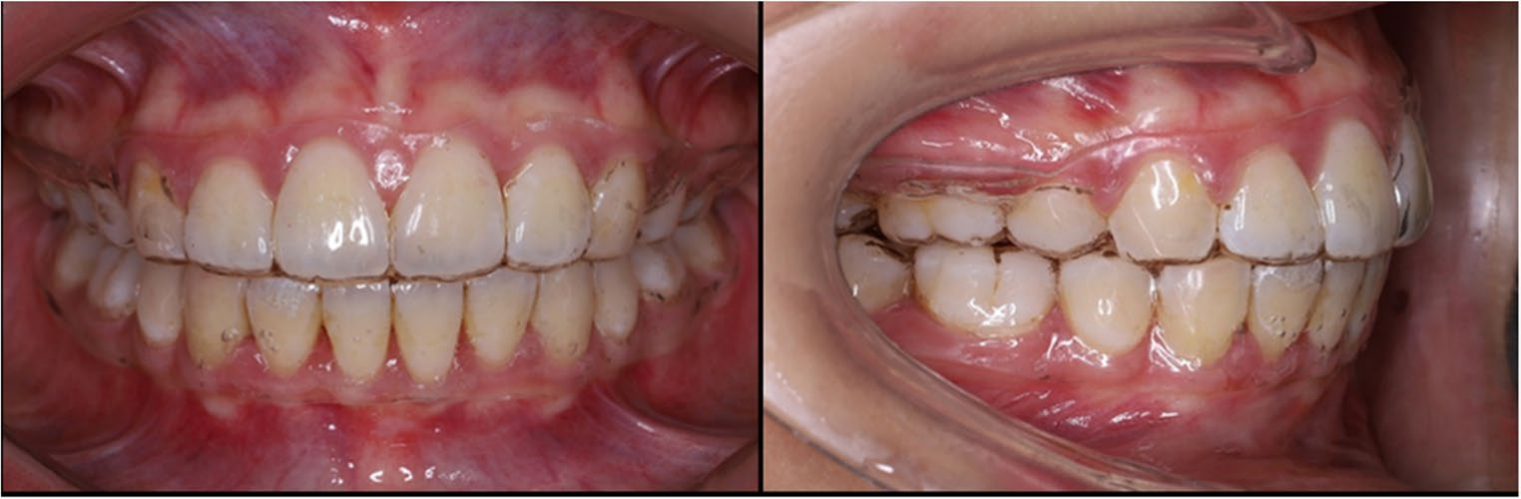
Thermoformed retainers (TFR) group

For the subjects in the TFR group (Fig. 1), an alginate impression (Hydrogum 5; Zhermack, Italy) was taken one-week post-debond for the construction of the TFR for research purposes. The working models were trimmed, and undercuts were blocked as necessary. The TFRs were fabricated from a 1.0 mm thick Erkodur blank (Erkodent^®^ Erich Kopp GmbH, Pfalzgrafenweiler, Germany) using the Erkopress ES-200E (Erkodent^®^ Erich Kopp GmbH, Pfalzgrafenweiler, Germany) machine following the manufacturer’s instructions. The TFRs were trimmed to provide 2 mm of gingival coverage (buccal/labial and palatal/lingual) and terminal molar three-quarter occlusal coverage. Finishing was performed using fine tungsten carbide burs (Scheu-Dental GmbH, Germany) and fine soft-brush burs (Edenta, Switzerland). All thermoformed retainers were fabricated in-house by a single operator following a standardised protocol.

**Fig. 2:**
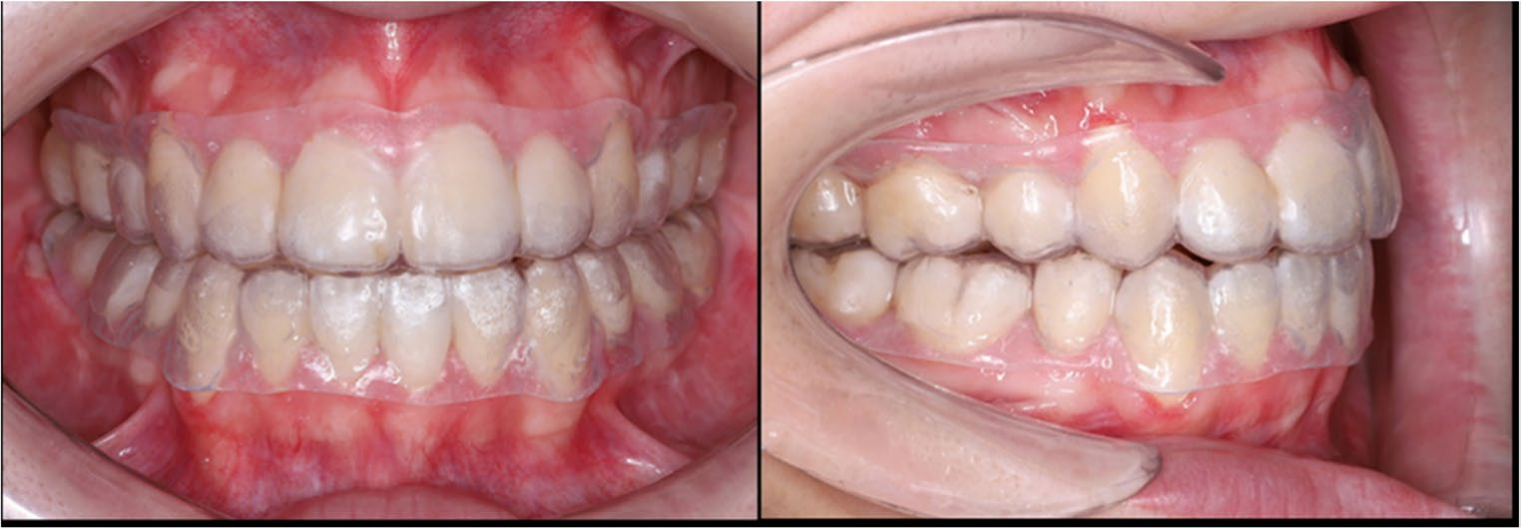
3D-printed retainers (3DPR) group

For subjects in the 3DPR group (Fig. 2), the dentition was scanned using the Trios 3 intraoral scanner (3Shape Trios A/S, Copenhagen, Denmark) one-week post-debond for baseline recording (T0) and the construction of digital models. The digital models were converted to Standard Tessellation Language (STL) files for retainer design. The principal investigator designed the retainers using 3Shape Design System-Splint Studio software (3Shape Unite 23.1, 3Shape A/S, Copenhagen, Denmark), ensuring an even thickness of 0.75 mm, coverage of all the dentition, up to 2 mm of the gingival margin (buccal/labial and lingual/palatal), terminal molar three-quarter occlusal coverage with an offset of 0.03 mm and undercuts were digitally blocked when necessary. After that, the STL files were sent to a private dental laboratory, where the 3DPRs were printed using NextDent Ortho Flex resin (NextDent, Vertex-Dental B.V., Soesterberg, The Netherlands). Prior to printing, the bottle was mixed with a NextDent LC-3DMixer (Vertex-Dental B.V., Soesterberg, The Netherlands) for five minutes. The retainers were printed using a NextDent 5100 3D printer (3D Systems, Vertex-Dental B.V., Soesterberg, The Netherlands) at 0 degrees, with a layer thickness of 100 μm and 405 nm blue-violet light. Post-printing, the retainers were washed twice in isopropyl alcohol (greater than 90%) for five minutes, dried with compressed air, and then post-cured in a UV-light curing box according to the manufacturer’s instructions. Supports were removed, and edges were smoothed using polishing burs. All direct 3D-printed retainers were fabricated by the same external digital laboratory using a single resin system and predefined design parameters, in accordance with manufacturer instructions.

Clinically, both 3DPRs and TFRs were issued within three weeks post-debond and evaluated by the principal investigator. All retainers underwent clinical inspection prior to delivery. The initial non-interventional TFRs were kept by the clinician in labelled retainer boxes. Participants were instructed to wear the retainers part-time (8–12 h daily) and store them in the provided retainer boxes when not in use. Standard-written wear-and-care instructions were provided. Compliance was monitored via monthly text reminders. Baseline (T0) measurements were recorded where dentition were scanned using Trios 3 intraoral scanner (3Shape Trios A/S, Copenhagen, Denmark) at the time of fitting of the assigned study retainers, which was at three weeks post-debond, rather than immediately after debonding. Subsequent measurements were obtained at three months (T1) and six months (T2).

### Outcome measures

The primary outcome measure was the stability of post-treatment following the use of TFR and 3DPR. The upper and lower dentition of subjects were scanned at two time points: T0 (baseline, during research retainer fitting), and T2 (6 months post-fitting) using the 3Shape Trios 3 intraoral scanner (3Shape Trios A/S, Copenhagen, Denmark). The measurements were performed digitally using the Patient Monitoring software in 3Shape Unite software (version 23.1, 3Shape A/S, Copenhagen, Denmark).

**Fig. 3:**
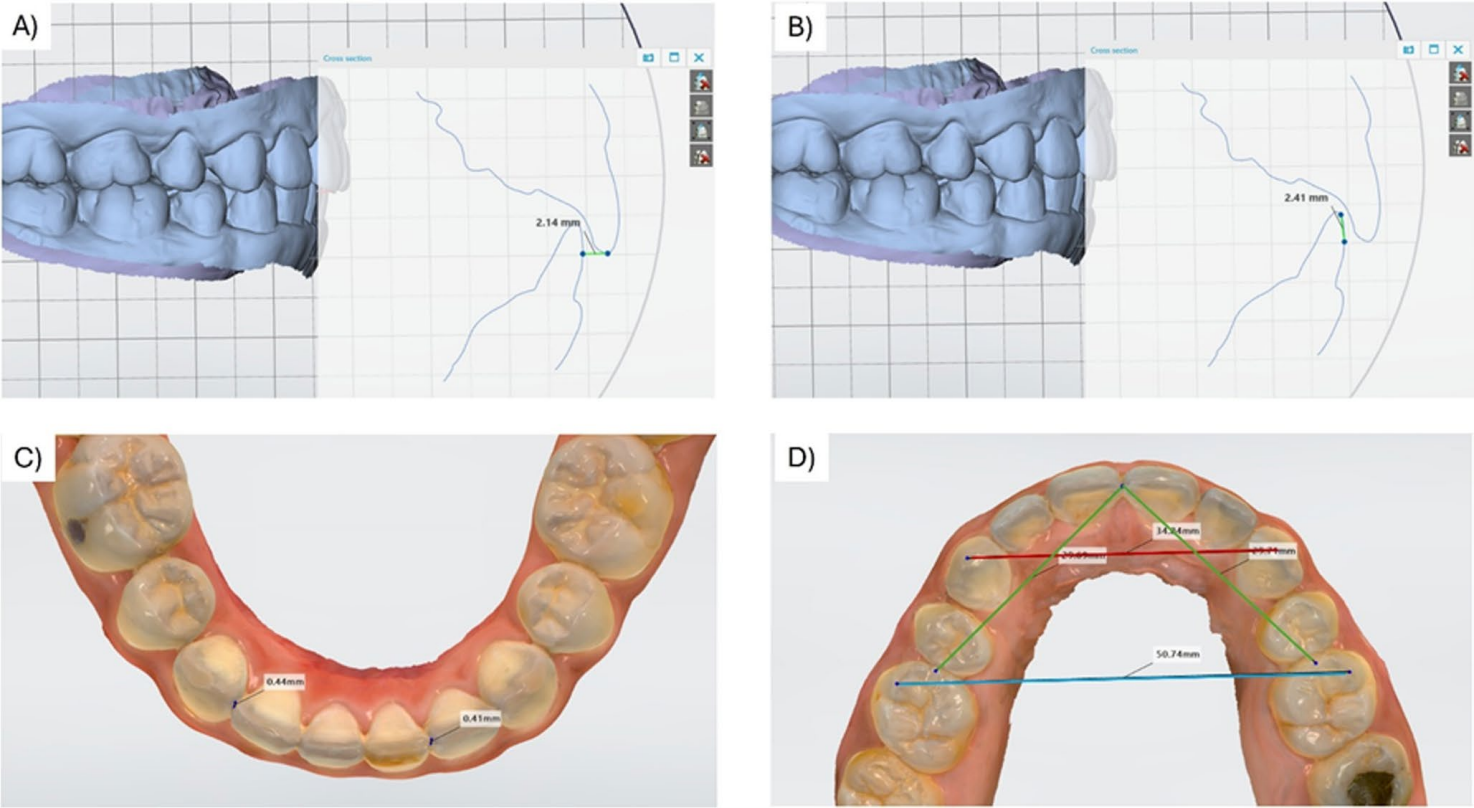
(**A**) Demonstration of measurement of overjet (OJ). (**B**) Demonstration of measurement of overbite (OB). (**C**) Demonstration of measurement of Little’s Irregularity Index (LII). (**D**) Demonstration of measurement of ICW (red line), IMW (blue line) and Arch length (sum of the distance of both green lines)

Scans from T0 and T2 were selected for evaluation. The software’s superimposition function was used to align all dental arches across these time points concurrently. The maxillary occlusal plane, defined as a plane passing through an idealized contact point between the upper central incisors and the mesiopalatal cusp tips of the first molars [28], served as the reference plane. Before measuring overjet (OJ) and overbite (OB), the upper arches were adjusted to this reference plane. Subsequently, a cross-sectional plane perpendicular to the reference plane was created. During the measurement of OJ and OB [Figure 3 (A and B)] at T0, dental arches from other time points (T2) were hidden to avoid any confusion. This process was repeated independently for T2 to ensure consistent standardized measurements. To assess the Little’s Irregularity Index (LII), intercanine width (ICW), intermolar width (IMW), and arch length (AL), the arches were viewed in three planes to ensure the landmarks were accurately located [(Fig. 3 (C and D)].

Patient’s OHRQoL was evaluated using a modified short version of the Oral Health Impact Profile-14 for Malaysian adults [OHIP-14(M)], a self-administered questionnaire tailored to include questions relevant to individuals wearing removable retainers [23, 24]. The questionnaire was administered to participants at two time points: T0 and T2. It consisted of seven domains, each containing two questions, resulting in a total of 14 questions. Responses to each question were scored on a five-point Likert scale, ranging from “never” (score 0) to “very often” (score 4).

### Blinding

This study was single-blinded, with the outcome assessor being blinded. Blinding was performed by NMT, who was unaware of the participants’ allocation to the retainer. The digital scans were reviewed, and OHIP-14[M] questionnaire data entry was performed by NMT, who then assigned a blinding code to each subject. After assigning the blinding code to each subject, BHS performed the outcome measurements. Group allocations were only revealed during statistical analysis.

## Statistical methods

### Error of the method

The intraclass correlation coefficient (ICC) was employed to assess intra-rater and inter-rater reliability for measuring LII, ICW, IMW, AL, OJ, and OB. The ICC values ranged from 0.0 to 1.0, with a value near 1 indicating high reliability among raters and a value near 0 indicating low reliability among raters [29]. To determine inter-examiner reliability, two investigators (NMT and BHS) used ten pairs of digital dentitions for calibration. For intra-examiner reliability, the principal investigator (BHS) remeasured these dentitions after a two-week interval. The measurements demonstrated excellent reliability, with inter-examiner ICC values ranging from 0.937 (95% Confidence interval [CI]: 0.841, 0.975) to 0.989 (95% CI: 0.965, 0.996) and intra-examiner ICC values ranging from 0.924 (95% CI: 0.808, 0.970) to 0.992 (95% CI: 0.980, 0.997) (Supplementary Files).

### Statistical analysis

Data analysis was conducted using IBM SPSS Statistics for Windows, Version 26.0 (IBM Corp, Armonk, NY, USA). The Shapiro–Wilk test was used to assess the normality of all continuous variables. As post-treatment stability outcomes and OHIP-14 scores were non-normally distributed, non-parametric tests were applied. Between-group comparisons were conducted at each time point using the Mann–Whitney U test. Within-group changes from baseline (T0) to 6 months (T2) were assessed using the Wilcoxon signed-rank test. Descriptive demographic variables were analysed using Fisher’s exact test or the Mann–Whitney U test, as appropriate. Statistical significance was set at *p* < 0.05. The data were analyzed using the intention-to-treat analysis. To address missing data, the last observation carried forward [30] was employed for the post-treatment stability. Maximum likelihood estimation [31, 32] was used for the OHIP-14 (M) data.

## Results

### Participants flow and recruitment

**Fig. 4:**
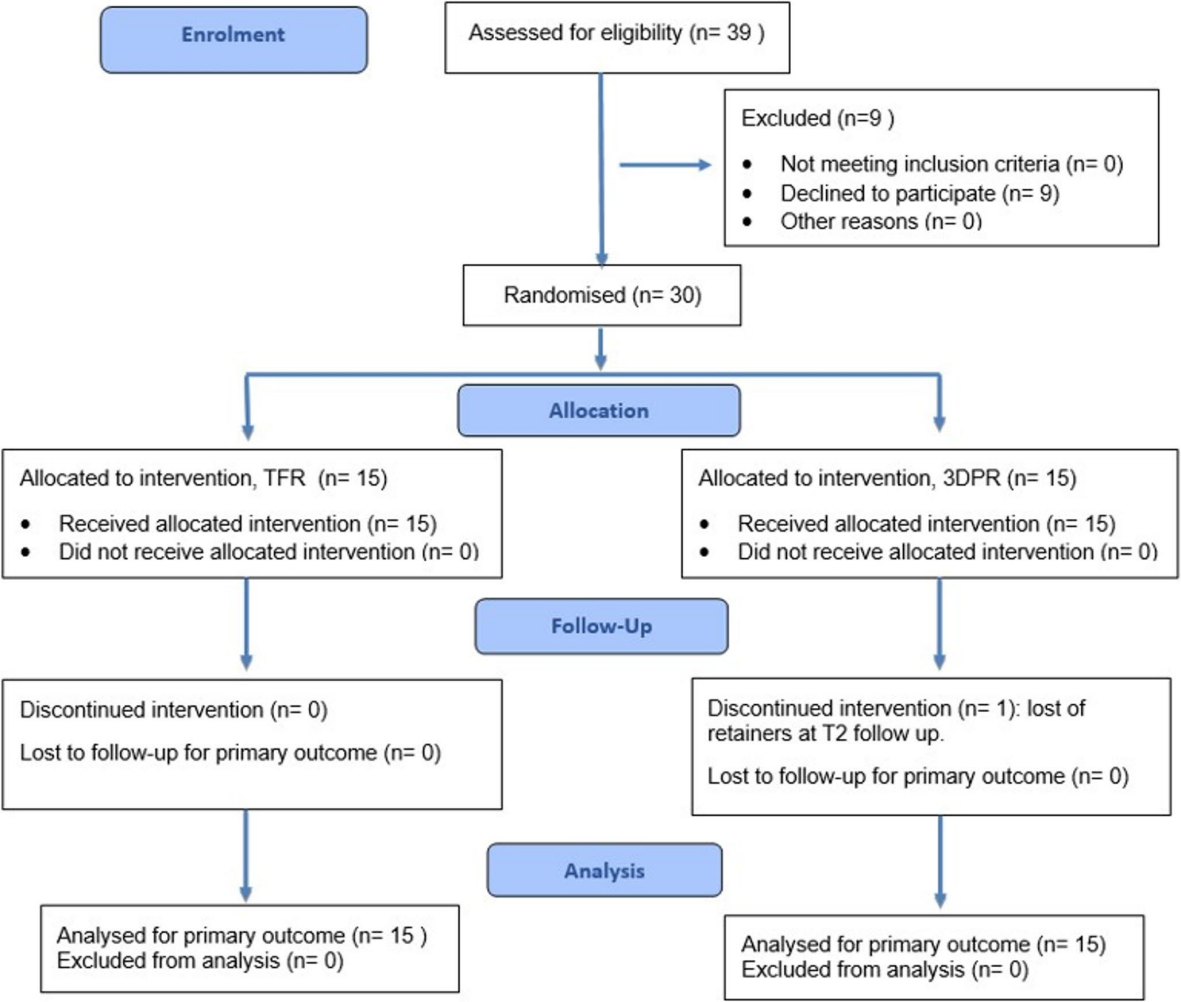
CONSORT 2025 flow diagram

Of the 39 patients who met the inclusion criteria for this study, 9 declined participation, primarily citing their inability to adhere to the required review schedule over a six-month period due to work and academic commitments. Consequently, a total of 30 patients were randomized into two groups: 15 in the TFR group and 15 in the 3DPR group. By the T2 assessment, 29 patients had completed the trial. The TFR group retained all 15 participants, whereas the 3DPR group experienced a dropout after T1 due to a participant accidentally losing her retainers, resulting in 14 participants. The CONSORT 2025 flow diagram is presented in Fig. 4.

### Baseline data

Table 1 presents the demographic data of the participants for the TFR and 3DPR groups. The total sample consisted of 30 participants, evenly divided between the two groups (15 participants per group). Both groups exhibited identical gender distributions (86.7% female and 13.3% male) and educational level distributions (86.7% tertiary and 13.3% secondary). Ethnicity was balanced overall, with 50% Malay and 50% Chinese. However, the TFR group had a higher proportion of Chinese (60%), while the 3DPR group had a higher proportion of Malay (60%), although the difference was not statistically significant (*p* = 0.466). The mean age in the TFR group was slightly higher at (23.47 ± 4.61 years) than in the 3DPR group (21.93 ± 4.17 years), but the difference was not statistically significant (*p* = 0.558).

**Table 1:** Baseline social demographic characteristics of both groups

Characteristics	Total *N* = 30 *n* (%)	TFR group, *n* = 15 *n* (%)	3DPR group, *n* = 15 *n* (%)	*P*-value^h, t^
Gender				
Male	4 (13.3%)	2 (13.3%)	2 (13.3%)	1.000^h^
Female	26 (86.7%)	13 (86.7%)	13 (86.7%)	
Ethnicity				
Malay	15 (50%)	6 (40%)	9 (60%)	0.466^h^
Chinese	15 (50%)	9 (60%)	6 (40%)	
Educational level				
Secondary	4 (13.3%)	2 (13.3%)	2 (13.3%)	1.000^h^
Tertiary	26 (86.7%)	13 (86.7%)	13 (86.7%)	
Age				
(mean ± SD)	22.70 ± 4.30	23.47 ± 4.61	21.93 ± 4.17	0.558^t^

^h^Fisher’s Exact test, ^t^Mann-Whitney U test

*p < 0.05 is significant; TFR: Thermoformed retainer 3DPR: 3D-printed retainer

### Primary outcome: post-treatment stability

Table 2 presents the post-treatment stability values, comparing measurements between the TFR and 3DPR groups at baseline (T0) and 6 months (T2). At baseline (T0), no significant differences were observed between the TFR and 3DPR groups across all upper and lower arch stability measures, except for overbite, which was greater in the TFR group (median 1.98 mm vs. 1.30 mm, *p* = 0.005). At 6 months (T2), both groups demonstrated similarly stable occlusal and arch dimensional characteristics, with no significant between-group differences in Little’s Irregularity Index (LII), intercanine width (ICW), intermolar width (IMW), or arch length (AL) in either arch. Overjet and overbite also remained comparable, although overbite continued to be higher in the TFR group (*p* = 0.022). Although the overbite is statistically significant between groups, the values were not clinically significant.

**Table 2:** Comparison of post-treatment stability values of TFR and 3DPR at baseline (T0) and six months (T2)

Variables	Time point	TFR group Median (IQR), mm	3DPR group Median (IQR), mm	*P*-value ^t^
Upper arch				
LII	**T0**	0.240 (0.600)	0.440 (0.930)	0.595
	**T2**	0.400 (0.830)	0.710 (1.030)	0.270
ICW	**T0**	37.160 (1.930)	36.610 (1.850)	1.000
	**T2**	37.160 (1.740)	36.620 (1.960)	1.000
IMW	**T0**	51.660 (2.180)	51.870 (2.390)	0.713
	**T2**	51.660 (2.430)	51.910 (2.600)	0.683
AL	**T0**	62.630 (2.180)	61.810 (3.950)	0.713
	**T2**	62.630 (2.170)	62.750 (4.010)	0.744
Lower arch				
LII	**T0**	0.350 (0.920)	0.220 (0.510)	0.174
	**T2**	0.540 (1.310)	0.310 (0.550)	0.070
ICW	**T0**	27.680 (1.950)	28.440 (2.020)	0.305
	**T2**	27.760 (2.270)	28.720 (2.300)	0.270
IMW	**T0**	43.070 (3.120)	42.480 (3.280)	0.838
	**T2**	43.120 (3.030)	42.670 (3.560)	0.983
AL	**T0**	52.340 (2.890)	52.160 (8.340)	0.950
	**T2**	52.390 (2.93)	52.160 (7.860)	0.950
In Occlusion				
OJ	**T0**	3.120 (1.040)	2.580 (2.160)	0.161
	**T2**	3.200 (1.100)	2.760 (1.580)	0.591
OB	**T0**	1.980 (1.070)	1.300 (0.720)	0.005*
	**T2**	2.050 (0.960)	1.350 (0.820)	0.022*

^**t**^ Mann-Whitney U test; *p < 0.05 is significant; TFR: Thermo-formed retainer; 3DPR: 3D-printed retainer

LII: Little’s Irregularity Index; ICW: Intercanine width; IMW: Intermolar width; AL: Arch length; OJ: Overjet; OB: Overbite

Table 3 indicates that the analysis of changes over the six-month period (Δ = T2 – T0) revealed no significant differences between groups for any variable (all *p* > 0.05). Hodges–Lehmann (HL) estimators indicated minimal median differences, with 95% confidence intervals crossing zero for all outcomes. Upper arch parameters demonstrated negligible change, including ΔLII (HL: 0.000 mm; 95% CI: − 0.260 to 0.000) and ΔICW (HL: − 0.040 mm; 95% CI: − 0.220 to 0.090). Similar findings were observed in the lower arch, where ΔLII (HL: 0.020 mm; 95% CI, 0.000 to 0.240) and ΔIMW (HL: − 0.020 mm; 95% CI, − 0.090 to 0.080) reflected small within-group changes that did not achieve between-group significance. Changes in overjet and overbite were likewise not different between groups (ΔOJ *p* = 0.089; ΔOB *p* = 0.745).

**Table 3:** Comparison of median changes (Δ = T2–T0) in post-treatment stability between TFR and 3DPR groups over six months

Variables	Changes	TFR group Median (IQR), mm	3DPR group Median (IQR), mm	HL estimate	95% CI (lower, upper)	*P*-value ^t^
Upper arch						
LII	**T2–T0**	0.000 (0.415)	0.020 (0.415)	0.000	(−0.260, 0.000)	0.314
ICW	**T2–T0**	0.050 (0.375)	0.190 (0.375)	−0.040	(−0.220, 0.090)	0.467
IMW	**T2–T0**	0.020 (0.090)	0.025 (0.090)	0.000	(−0.060, 0.090)	0.835
AL	**T2–T0**	0.040 (0.080)	0.000 (0.190)	0.020	(−0.040, 0.080)	0.382
Lower arch						
LII	**T2–T0**	0.150 (0.132)	0.000 (0.130)	0.020	(0.000, 0.240)	0.202
ICW	**T2–T0**	0.040 (0.315)	0.080 (0.315)	−0.050	(−0.220, 0.040)	0.245
IMW	**T2–T0**	0.050 (0.137)	0.055 (0.130)	−0.020	(−0.090, 0.080)	0.455
AL	**T2–T0**	0.020 (0.150)	0.000 (0.170)	0.010	(−0.050, 0.080)	0.663
In Occlusion						
OJ	**T2–T0**	0.080 (0.785)	0.290 (0.785)	−0.210	(−0.510, 0.040)	0.089
OB	**T2–T0**	0.060 (0.390)	0.050 (0.390)	−0.020	(−0.020, 0.130)	0.745

^**t**^ Mann-Whitney U test; *p < 0.05 is significant; HL = Hodges–Lehmann estimator of median difference; TFR: Thermo-formed retainer; 3DPR: 3D-printed retainer

LII: Little’s Irregularity Index; ICW: Intercanine width; IMW: Intermolar width; AL: Arch length; OJ: Overjet; OB: Overbite

### Secondary outcome: OHRQoL of patients wearing 3DPR and TFR

Table 4 presents the OHIP-14 (M) scores across various domains at T0 and T2 for the TFR and 3DPR groups. At baseline (T0), there were no significant differences between the TFR and 3DPR groups in any OHIP-14 domain or in total scores (*p* > 0.05). At 6 months (T2), the groups remained comparable, except for psychological disability, where the 3DPR group reported slightly higher scores than the TFR group (*p* = 0.020).

Within-group analysis revealed significant improvements in several domains for both groups. The TFR group showed improvement in functional limitation (*p* = 0.022), psychological discomfort (*p* = 0.006), and total OHIP-14 score (*p* = 0.016). The 3DPR group exhibited significant improvements in functional limitation (*p* = 0.012), physical pain (*p* = 0.012), psychological discomfort (*p* = 0.003), and total OHIP-14 score (*p* = 0.013). Overall, both groups demonstrated improvements in OHRQoL over the six months, as reflected by the reduced OHIP-14 scores.

**Table 4:** OHIP-14 (M) scores for each domain and total scores at T0 and T2 for TFR and 3DPR groups

Domain	Time point	TFR Median (IQR)	3DPR Median (IQR)	*p*-value^t^ Between groups
Functional limitation	**T0**	3.00 (2.00)	3.00 (2.00)	0.848
	**T2**	2.00 (2.00)	2.00 (2.00)	0.535
	**p-value** ^**f**^ within group	0.022*	0.012*	
Physical pain	**T0**	1.87 (2.00)	2.00 (2.00)	0.523
	**T2**	1.00 (2.00)	1.00 (1.00)	0.493
	**p-value** ^**f**^ within group	0.077	0.012*	
Psychological discomfort	**T0**	2.00 (2.00)	2.00 (2.00)	0.714
	**T2**	1.00 (1.00)	1.00 (1.00)	0.620
	**p-value** ^**f**^ within group	0.006*	0.003*	
Physical disability	**T0**	1.00 (2.00)	2.00 (3.00)	0.608
	**T2**	1.00 (1.00)	1.00 (2.00)	0.965
	**p-value** ^**f**^ within group	0.204	0.176	
Psychological disability	**T0**	1.00 (2.00)	0.00 (3.00)	0.861
	**T2**	0.00 (0.00)	1.00 (2.00)	0.020*
	**p-value** ^**f**^ within group	0.055	0.776	
Social disability	**T0**	0.00 (2.00)	1.00 (1.00)	0.450
	**T2**	0.00 (0.00)	0.00 (1.00)	0.068
	**p-value** ^**f**^ within group	0.194	0.595	
Handicap	**T0**	0.00 (0.00)	0.00 (1.00)	0.065
	**T2**	0.00 (0.00)	0.00 (1.00)	0.141
	**p-value** ^**f**^ within group	1.000	0.336	
Total OHIP-14 scores	**T0**	9.00 (10.00)	11.0 (13.00)	0.819
	**T2**	4.00 (5.00)	7.00 (7.00)	0.219
	**p-value** ^**f**^ within group	0.016*	0.013*	

TFR: Thermoformed retainer; 3DPR: 3D-printed retainer

^**f**^ Wilcoxon Signed-Rank Test; ^**t**^ Mann-Whitney U test; *p < 0.05 is significant

### Harms

No serious harm was observed.

## Discussion

### Primary outcomes: Post-treatment stability

This study focused on clear removable retainers, despite being made from different materials, which share a similar design. Most existing research on the effectiveness of removable retainers primarily compares designs that differ significantly, such as TFR versus Hawley retainers or Begg retainers [27, 33–35]. Therefore, direct comparisons with these previous studies are challenging due to the design variations.

The findings of this study indicated no significant differences in post-treatment stability between TFR and 3DPR for LII, ICW, IMW, AL, and OJ at 3 and 6 months, except for OB. Consequently, the null hypothesis for the primary objective was not rejected for most of the parameters, except for OB. Overall, both TFR and 3DPR retainers maintained comparable post-treatment stability over a six-month period, with no clinically meaningful differences detected between the retainer types.

There were limited studies comparing the post-treatment stability of clear removable retainers made from different materials. Our findings align with those of Cunning et al. (2022), who conducted a prospective clinical trial to evaluate the effectiveness of two types of TFR, polyurethane-based and Polyethylene Terephthalate Glycol (PETG)-based, over a six-month period following clear aligner treatment. Their results indicated no statistically significant differences in changes in LII between the polyurethane and PETG groups for both the maxillary and mandibular arches at either the three-month or six-month follow-up points [36]. Similarly, our study demonstrated that TFR made of PETG and 3DPR made of photopolymerisable polymethylmethacrylate (PPMMA) resin exhibit comparable effectiveness in maintaining post-treatment stability.

In the present study, a significant difference in OB was observed between the TFR group and the 3DPR group at all time points. This discrepancy can be attributed to the baseline distribution of cases, in which the TFR group initially included subjects with a slightly higher OB, a condition that persisted throughout the six-month retention period. Given that the between-group difference in ΔOB was negligible and non-significant, the persistent statistical difference at T0 and T2 appears to be driven by this initial imbalance rather than by differential retainer performance. The OB of the TFR group remained within the average range of 2–4 mm at all time points [37], while the 3DPR group showed a slight reduction. Moreover, the median change in OB over six months was minimal and almost identical between groups (0.06 mm in TFR vs. 0.05 mm in 3DPR; HL −0.02 mm, 95% CI −0.02 to 0.13; *p* = 0.745), indicating that the retainers themselves did not differentially influence overbite.

Thickett and Power (2010) conducted a one-year follow-up study and reported that most parameters, including LII, ICW, IMW, arch length, and overjet, showed no significant differences between part-time and full-time wear of TFR at all time points. However, they noted a significant increase in OB in the part-time wear group at both the six-month and one-year reviews. They suggested that part-time wear of TFR facilitates more rapid settling compared with full-time wear [38]. Consistent with their findings, the present study also observed an increase in OB during the 6-month follow-up period. However, this increase was not clinically significant. Schutz-Fransson et al. (2006), in their long-term stability study spanning up to 11 years, found that a small increase in OB (0.8 mm) following the completion of orthodontic treatment is a common finding in long-term studies [39]. Furthermore, the median change in OB between the TFR and 3DPR groups was not significant, potentially due to similar rates of occlusal settling in both groups.

Participants in this study were instructed to wear their retainers part-time for 8 to 12 h daily over a 6-month research period. Gill et al. (2007) investigated the effectiveness of part-time versus full-time wear of TFR and found no significant differences in LII, ICW, IMW, OJ, or OB between the two groups from the end of treatment to six months post-retention. The authors suggested that nighttime wear of TFR was as effective as full-time wear in maintaining dental alignment and occlusion stability [22]. Comparing the findings of Gill et al. (2007) with those of the present study suggests that wearing either TFR or 3DPR part-time over six months effectively maintains occlusion, with post-treatment changes being statistically insignificant.

### Secondary outcomes: OHRQoL of patients wearing 3DPR and TFR

The findings of this study suggest that both TFR and 3DPR have minimal long-term impact on patients’ OHRQoL when used under a part-time wear protocol. A small but statistically significant difference between groups was detected in the psychological disability domain at T2, with the 3DPR group reporting marginally higher scores. Although the magnitude of this difference was modest, it may reflect the increased material thickness typically associated with 3D-printed retainers. Unlike thermoformed retainers, which undergo predictable thinning during the thermoforming process [40], 3D-printed retainers are produced layer by layer and may retain a greater uniform thickness or exhibit localised increases [16, 41, 42]. Increased bulk may heighten awareness of the appliance during wear, potentially contributing to perceptions of inconvenience or a mild psychological burden, as captured by this domain of the OHIP-14.

Despite this isolated finding, no significant differences in total OHIP-14 scores were observed between groups at either time point. Both groups showed significant reductions in OHIP-14 scores from T0 to T2, indicating improvements in oral health-related quality of life over the 6-month period. This pattern aligns with previous studies, such as Monk et al. (2021), who reported improvements in OHRQoL following orthodontic appliance removal, attributed to enhanced aesthetics, functional comfort, and the alleviation of treatment-related discomfort [26]. The higher baseline OHIP-14 scores in this study compared with other studies [24–26] may reflect participants already wearing their non-interventional TFR for several weeks before switching to the research retainers. During this period, participants were still adapting to part-time retainer wear, which may have transiently elevated their perceived impacts on daily activities. By 6 months, participants in both groups appeared fully accustomed to their retainers, and the reduced OHIP-14 scores suggest that long-term wear imposed minimal disruption to daily life.

### Limitations

This single-centre RCT may be subject to operator-related bias, which could affect the generalizability of the findings. In addition, the small sample size reduces statistical power, warranting larger future studies to validate the results. Blinding of the operator and participants was unfeasible due to visible differences between TFR and 3DPR. Furthermore, 3DPR fabrication relies on external laboratory, disrupting clinical workflows and delaying the issuance of retainers. To standardise the timing of retainer issuance between groups and to prevent post-debond relapse, a short-term transitional retainer (non-interventional TFR) was provided, as it was deemed unethical to leave patients without retainers. Both groups wore this non-interventional TFR for three weeks post-debond, and as this protocol was applied uniformly, it is unlikely to have influenced between-group comparisons. The six-month follow-up may be insufficient to assess the long-term efficacy of the retainer, suggesting the need for extended observation periods. Lastly, compliance was assessed indirectly through standardised instructions and monthly reminders, without objective wear-time monitoring. Although this reflects common clinical practice, future studies incorporating compliance sensors may provide more detailed insight into retainer-wearing behaviour.

### Generalizability

The generalizability of this study is limited to the short-term retention phase, with a six-month follow-up. The generalizability is inherently linked to the performance of 3DPR fabricated using NextDent Ortho Flex resin in young adult patients.

## Conclusion

1. There were no significant differences between TFR and 3DPR in terms of LII, ICW, IMW, AL, and OJ across all time points (T0 and T2) except for OB.
2. Post-treatment stability showed no significant median differences for all the parameters between TFR and 3DPR during the overall six-month period (T0-T2).
3. In most OHIP-14 (M) domains, there were no significant median differences between TFR and 3DPR, except in the psychological disability domain at T2, where 3DPR scored significantly higher than TFR. Both groups showed improvements in OHRQoL.

## Supplementary Information

Below is the link to the electronic supplementary material.

Supplementary Material 1 (DOCX 21.1 KB)

## Data Availability

Data is available upon request from the corresponding author.
