# Supplementary material for: A Randomised Controlled Trial Comparing Thermoformed and 3D-Printed Retainers in Young Adults: Evaluation of Post-treatment Stability and Patient Satisfaction
Source: Clin Oral Investig. 2026 Mar 17;30(4):126. doi: 10.1007/s00784-026-06793-z (PMC12992356; doi:10.1007/s00784-026-06793-z)
Supplement: Supplementary file 1 — Supplementary Material 1 (DOCX 21.1 KB) [file 784_2026_6793_MOESM1_ESM.docx]

SUPPLEMENTARY TABLES

**Table S1: Brief defintion and description of the surface roughness parameters**

| **Parameters** | **Unit** | **Description** |
| --- | --- | --- |
| Ra | μm | Arithmetic mean roughness. Average roughness represents the arithmetic mean of the surface texture, with the valleys inverted to achieve a positive value |
| Rq | μm | RMS roughness. Root mean square average of the profile heights over the evaluation length |

**Table S2: Definition of the Mechanical Properties Tested**

| Mechanical Properties (Unit) | Definition |
| --- | --- |
| Martens hardness HM (MPa or N/mm^2^) | Martens hardness is defined as the test force (F) divided by the surface area (As(h)) of the indenter in contact with the specimen at a given indentation depth (h).  It reflects the material's resistance to elastic and plastic deformation under load.  *Formula: HM= F/As(h)*  Where:  F: Test force  As (h): The surface area of the indenter at distance *h* from the tip. |
| Elastic Index ƞIT (%) | The elastic index represents the ratio of elastic deformation work (Welast) to the total mechanical work (Wtotal) performed during indentation.  It indicates the material's ability to recover elastically after unloading.  *Formula: ƞIT = Welast / Wtotal × 100*  Where:  *W*total: Total mechanical work of indentation  *W*elast: Elastic reverse deformation work of indentation. |
| Indentation Modulus EIT (GPa) | Indentation modulus is calculated from the slope of the unloading curve at maximum load during indentation.  It approximates the material's Young’s modulus, reflecting its stiffness, rigidity or resistance to elastic deformation. |
| Indentation Hardness HIT (MPa) | Indentation hardness measures a material's resistance to permanent deformation or damage.  It is defined as the maximum applied force (Fmax) divided by the projected contact area (Ap) between the indenter and the specimen.  *Formula: HIT = Fmax / Ap*  Where:  *F*max: The maximum applied force;  *A*p: The projected (cross-sectional) area of contact between the indenter and the test piece determined from the force displacement curve and a knowledge of the area function of the indenter. |

**Table S3: Surface roughness parameters (Ra and Rq) of thermoformed retainers (TFR) and direct 3D-printed retainers (3DPR) at control and after intraoral use in the upper and lower arches**

| Arch | Surface roughness | Time point | TFR group  Median (IQR), µm | | 3DPR group  Median (IQR) , µm | | Among-group p-value,^k^ |
| --- | --- | --- | --- | --- | --- | --- | --- |
|  |  |  | Cameo  (n=5) | Intaglio  (n=5) | Cameo  (n=5) | Intaglio  (n=5) |  |
| Upper | Ra | Control | 0.20 (0.05) | 0.42 (0.43) | 0.48 (0.65) | 1.57 (0.73) | 0.003* |
|  |  | Used | 0.18 (0.11) | 0.44 (0.48) | 0.63 (0.78) | 1.08 (1.67) | 0.018* |
|  |  | Within-group p-value, ^w^ | 0.47 | 0.89 | 0.35 | 0.08 |  |
|  | Rq | Control | 0.23 (0.04) | 0.49 (0.54) | 0.74 (0.9) | 2.27 (0.88) | 0.003* |
|  |  | Used | 0.22 (0.11) | 0.52 (0.6) | 0.78 (0.89) | 1.38 (1.96) | 0.015* |
|  |  | Within-group p-value, ^w^ | 0.47 | 0.89 | 0.35 | 0.08 |  |
| Lower | Ra | Control | 0.17 (0.07) | 0.23 (0.28) | 0.43 (0.89) | 1.97 (2.16) | 0.015* |
|  |  | Used | 0.15 (0.29) | 0.29 (0.29) | 0.49 (1.84) | 1.49 (1.52) | 0.009* |
|  |  | Within-group p-value, ^w^ | 0.79 | 0.89 | 0.69 | 0.35 |  |
|  | Rq | Control | 0.22 (0.07) | 0.28 (0.31) | 0.61 (1.19) | 2.32 (2.60) | 0.012* |
|  |  | Used | 0.18 (0.32) | 0.35 (0.38) | 0.58 (2.07) | 2.07 (1.91) | 0.007* |
|  |  | Within-group p-value, ^w^ | 0.50 | 0.69 | 0.89 | 0.35 |  |

Used: After 6 months of intraoral use; w: Wilcoxon signed-rank test; k: Kruskal–Wallis test; *Statistical significance was set at p < 0.05

**Table S4: Post-hoc pairwise comparisons of surface roughness parameters (*Ra* and *Rq*) among retainer groups in the upper and lower arch using the Mann–Whitney U test**

| Arch | Comparison | Ra (Exact p value) | | Rq (Exact p value) | |
| --- | --- | --- | --- | --- | --- |
|  |  | Control | Used | Control | Used |
| Upper | TFR–Cameo vs TFR–Intaglio | 0.009 | 0.016 | 0.009 | 0.016 |
|  | TFR–Cameo vs 3DPR–Cameo | 1.000 | 0.016 | 1.000 | 0.008† |
|  | TFR–Cameo vs 3DPR–Intaglio | 0.008† | 0.016 | 0.008† | 0.016 |
|  | TFR–Intaglio vs 3DPR–Cameo | 1.000 | 1.000 | 0.841 | 0.690 |
|  | TFR–Intaglio vs 3DPR–Intaglio | 0.008† | 0.310 | 0.008† | 0.310 |
|  | 3DPR–Cameo vs 3DPR–Intaglio | 0.008† | 0.310 | 0.008† | 0.421 |
| Lower | TFR–Cameo vs TFR–Intaglio | 0.056 | 0.222 | 0.032 | 1.151 |
|  | TFR–Cameo vs 3DPR–Cameo | 0.151 | 0.056 | 0.151 | 0.056 |
|  | TFR–Cameo vs 3DPR–Intaglio | 0.008† | 0.008† | 0.008† | 0.008† |
|  | TFR–Intaglio vs 3DPR–Cameo | 0.421 | 1.151 | 0.310 | 0.095 |
|  | TFR–Intaglio vs 3DPR–Intaglio | 0.032 | 0.008† | 0.032 | 0.008† |
|  | 3DPR–Cameo vs 3DPR–Intaglio | 0.151 | 0.421 | 0.151 | 0.310 |

† Statistically significant at Bonferroni-adjusted α = 0.05/6 = **0.008**
